# Supplementary figures and images for: Stonewall prevents expression of ectopic genes in the ovary and accumulates at insulator elements in D. melanogaster
Source: PLoS Genet. 2022 Mar 24;18(3):e1010110. doi: 10.1371/journal.pgen.1010110 (PMC8982855; doi:10.1371/journal.pgen.1010110)

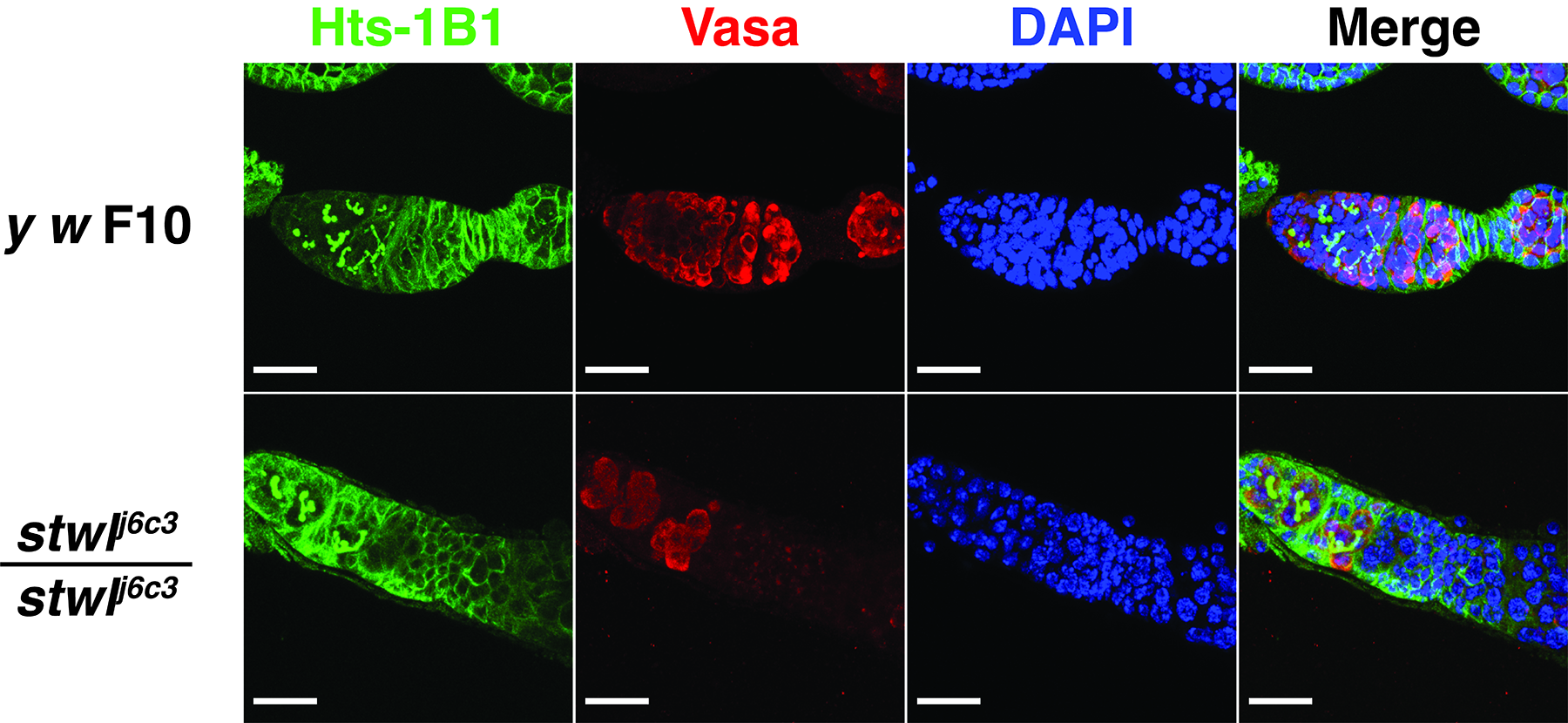

Supplement: S1 Fig — Ovaries were dissected from females of the indicated genotype 3–6 days post-eclosion. stwl null ovaries typically lack germ cells or contain severely disordered germline cysts [11–13]. ɑ-Vasa labels germ cells, ɑ-Hts-1B1 labels branched fusomes or spectrosomes as well as follicle cell membranes. Germaria are positioned with anterior to posterior going (left to right). stwl+ ovaries (y w F10) contain self-renewing GSCs (anterior-most germ cells) which differentiate into cystoblasts and become ordered, organized germline cysts. All images are maximum-intensity projections from a z-series representing a depth of 10 microns. Scale bars are 20 microns. (TIF) [file pgen.1010110.s001.tif]

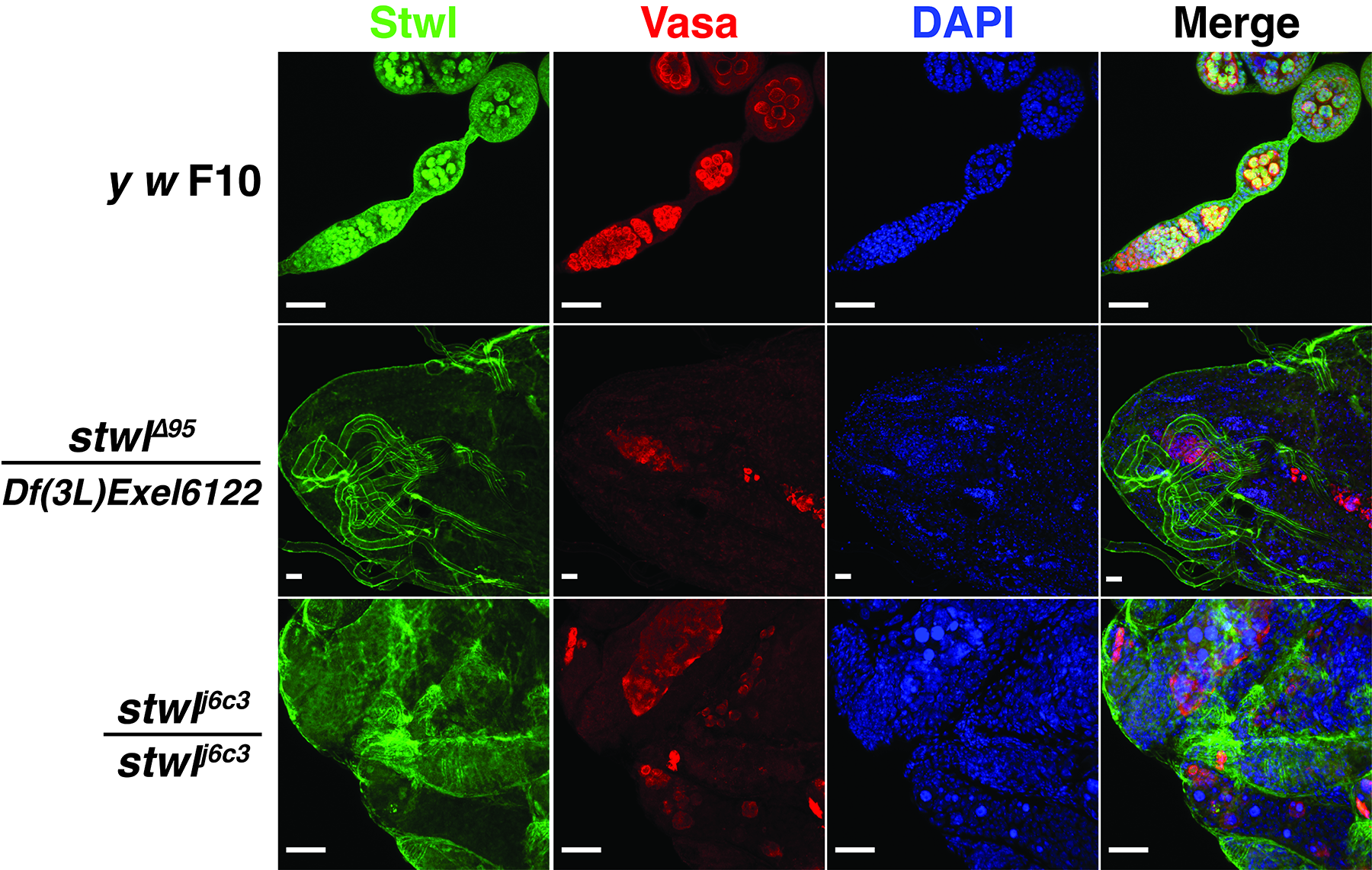

Supplement: S2 Fig — D. melanogaster ovaries were dissected from females 10–15 days post-eclosion, immunostained with ɑ-Stwl sera from GP 76 (Methods). ɑ-Vasa labels germ cells, which are typically not retained in older mutant ovaries. Germaria are positioned with anterior to posterior going left to right. All images are maximum-intensity projections from a z-series representing a depth of 10 microns. In stwl mutant images, the green channel is overexposed to demonstrate the absence of Stwl signal. Scale bars are 20 microns. The stwlΔ95/Df(3L)Exel6122 ovary (middle row panels) is displayed at 0.4x magnification relative to other images, to demonstrate loss of germline across the ovary. (TIF) [file pgen.1010110.s002.tif]

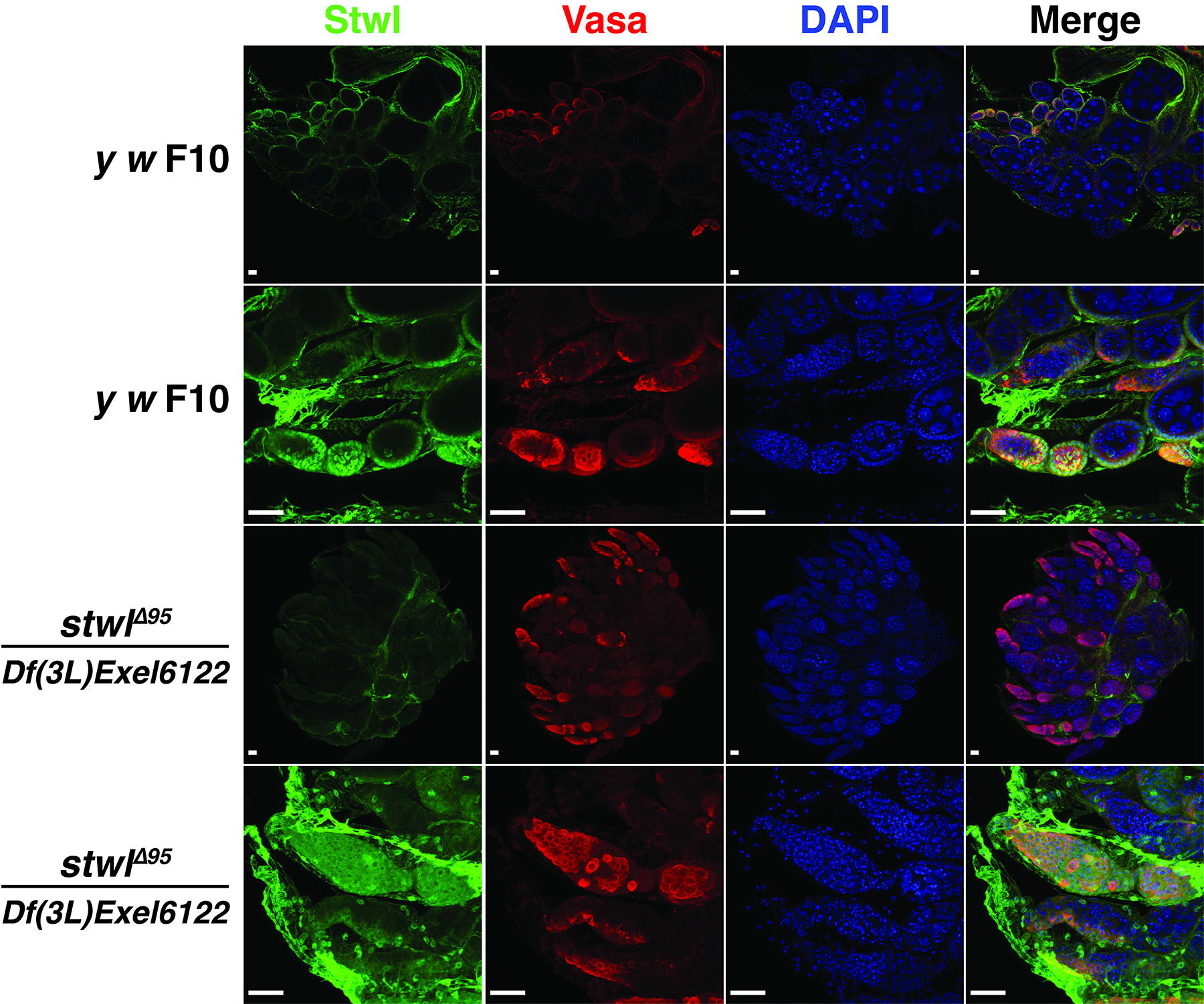

Supplement: S3 Fig — D. melanogaster ovaries were dissected from females <12-hours post-eclosion and immunostained with ɑ-Stwl sera from GP 76 (Methods). ɑ-Vasa labels germ cells, which are typically not retained in older mutant ovaries. Germaria are positioned with anterior to posterior going left to right. Wild-type ovaries produce egg chambers up to stage 7 or 8, while stwl mutant ovaries maintain egg chambers up to about stage 6 or 7. Low (0.24x) magnification images are of a single confocal slice (first and third rows), higher magnification images are maximum-intensity projections from a z-series representing a depth of 10 microns (second and fourth rows). In stwl mutant images, green channel is overexposed to demonstrate absence of Stwl signal. (TIF) [file pgen.1010110.s003.tif]

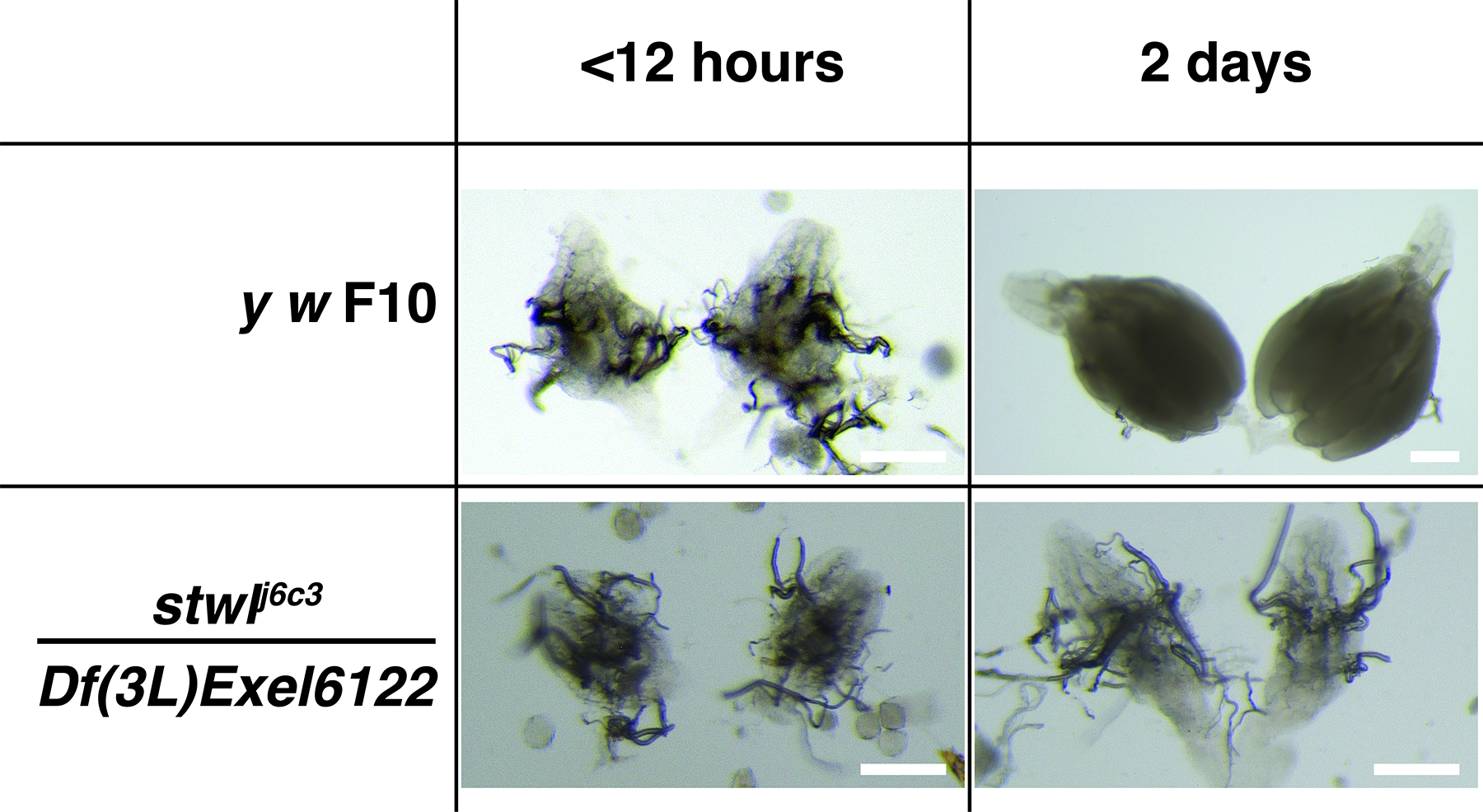

Supplement: S4 Fig — Ovaries were dissected from newly-eclosed and two-day-old females of the indicated genotypes. stwl deficient ovaries are rudimentary, but more closely resemble wild-type ovaries when from newly-eclosed individuals. Scale bars are 1 mm. (TIF) [file pgen.1010110.s004.tif]

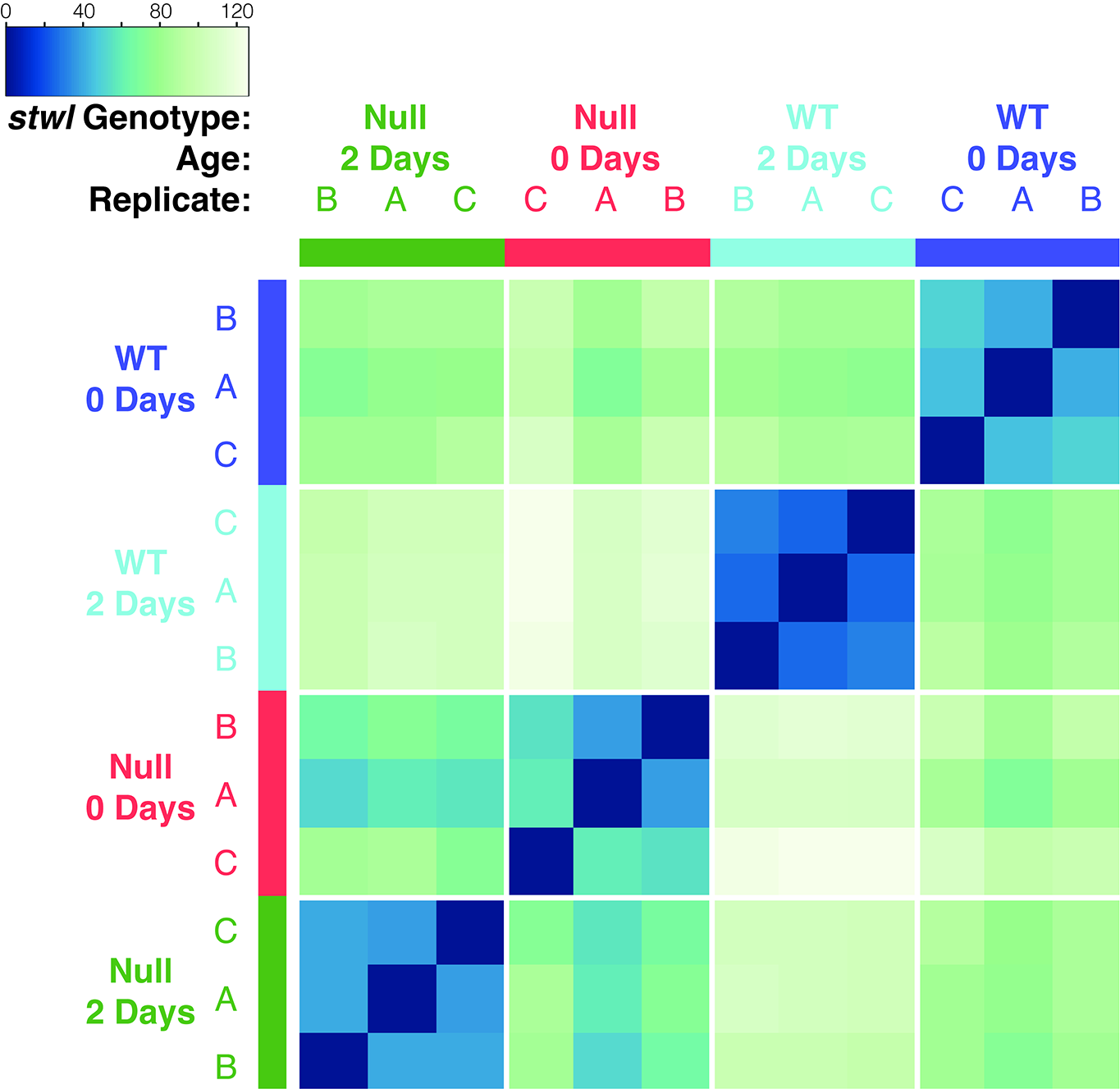

Supplement: S5 Fig — Read counts were regularized log transformed in DESeq, and the distance between samples was calculated based on these transformed count values. The heatmap is sorted by similarity after hierarchical clustering and color-coded according to distance, where dark blue cells indicate a distance of 0 (completely self-similar) and white cells a maximal distance (completely dissimilar). Samples within the same group (identical age and genotype) occur together and form blue clusters. (TIF) [file pgen.1010110.s005.tif]

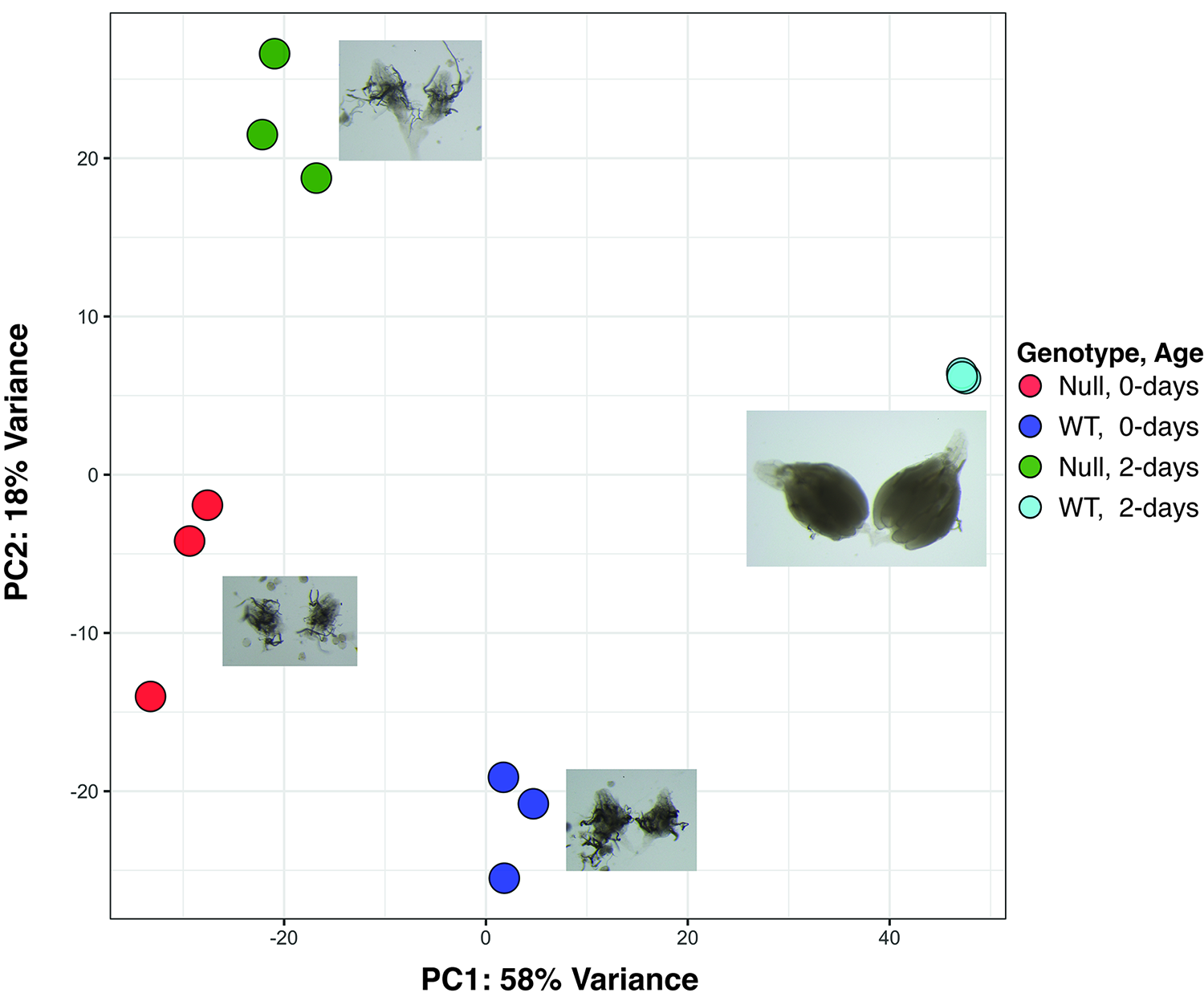

Supplement: S6 Fig — PCA was performed on regularized log transformed read counts of the 500 most variable genes in the count matrix. Samples within the same group (identical age and genotype) cluster together, indicating minimal batch effects. (TIF) [file pgen.1010110.s006.tif]

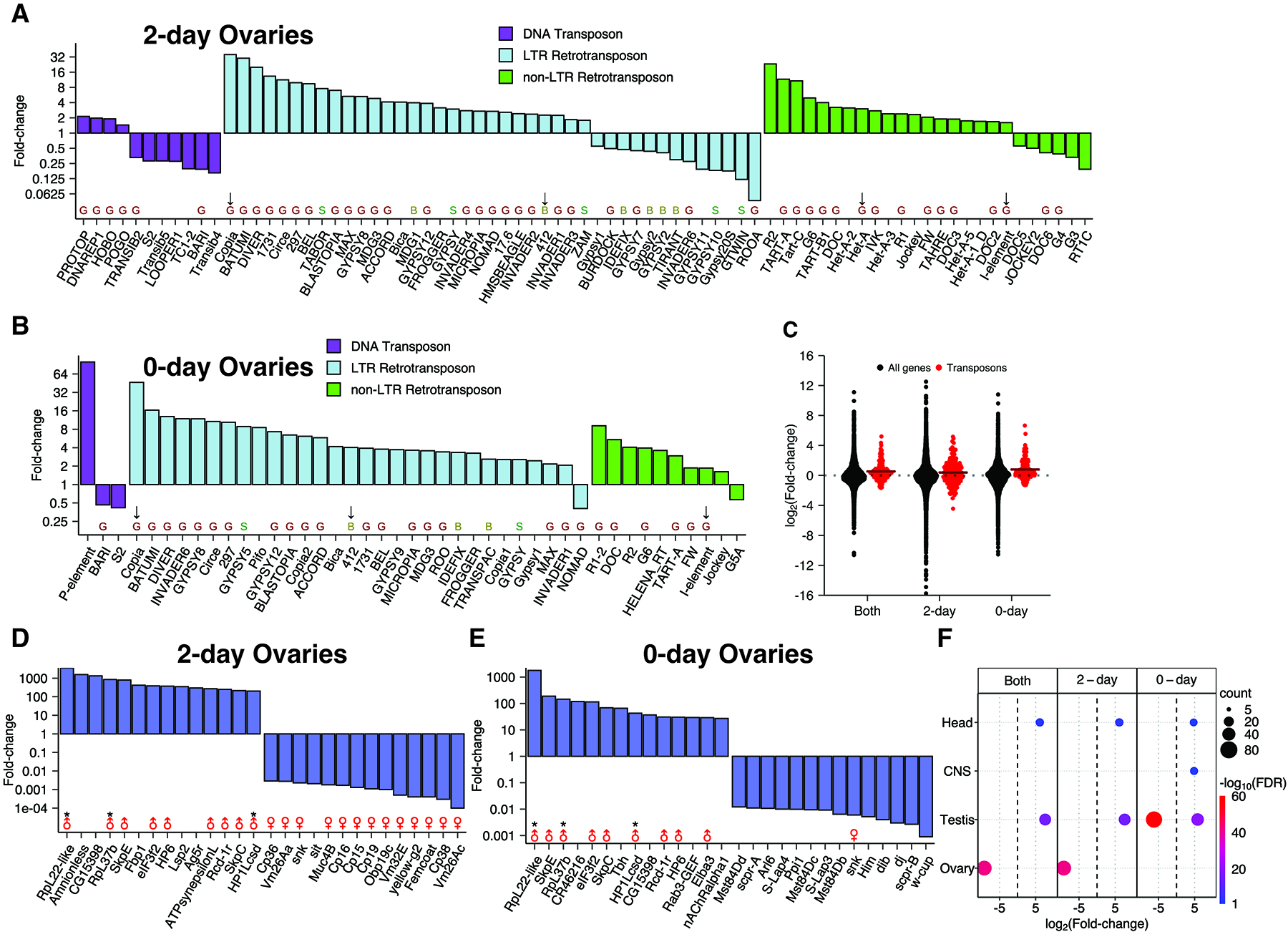

Supplement: S7 Fig — (A-B) Fold-change of TEs in stwl null (stwlj6c3/stwlj6c3) relative to wild-type from RNA-Seq assay of 0- and 2-day old ovaries. Black arrows point to TEs validated with qRT-PCR data in Fig 1A and/or 1B. “G”,”S”,”B” indicates whether TE is typically expressed in germline, ovarian soma, or both, respectively [26]. (C) log2Fold-change (LFC) of TEs vs. all genes from stwl null ovaries in the combined GLM, 2-day, and 0-day datasets, relative to wild-type. Crossbars show the mean LFC for all TEs. (D-E) Fold-change of the top 14 and bottom 14 most affected annotated genes (based on FlyBase annotations) in stwl null ovaries relative to wild-type. Male and female symbols mark genes with testis- and ovary-enriched wild-type expression, respectively; “*” marks genes that are part of the 59C4-59D testis-specific cluster described in Fig 2. (F) Enriched tissue classes among the top and bottom 1% of misregulated genes. Average LFC is plotted for each set of tissue-enriched genes enriched among stwl null ovaries relative to wild-type. Only gene sets with FDR <0.05 are plotted. (TIF) [file pgen.1010110.s007.tif]

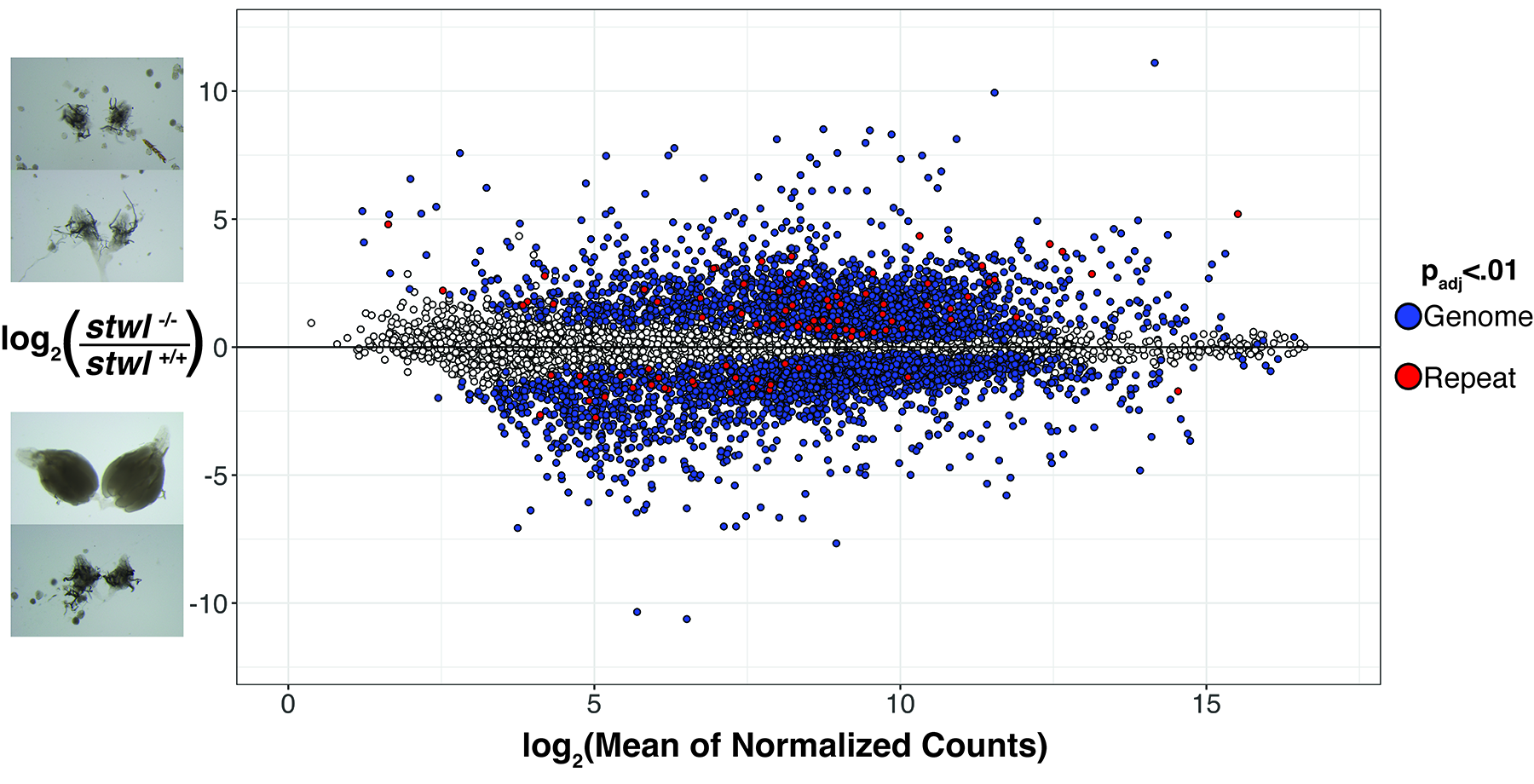

Supplement: S8 Fig — Fold-change for each gene is plotted against its average transcript abundance across all assayed ovarian samples (wild-type and null). Transcript abundance is represented by counts normalized according to GC-content and library size. The log2(Fold-change) values (LFC) were “shrunk” to minimize the variance associated with low-count genes. Filled points (blue and red) identify genes which are differentially expressed (adjusted p-value <0.01) in this comparison. Red points represent entries from Repbase, blue points are from the genomic annotation. (TIF) [file pgen.1010110.s008.tif]

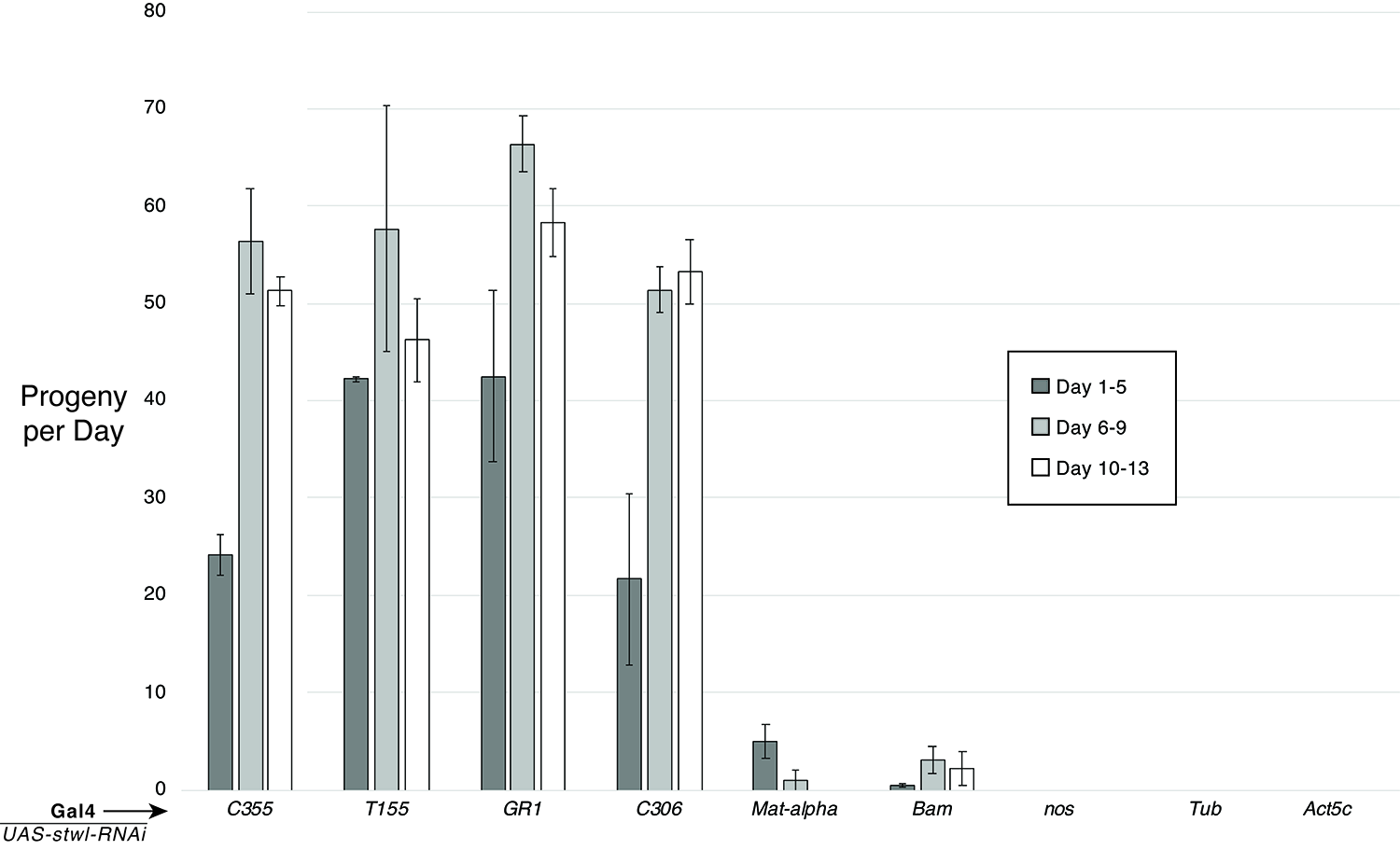

Supplement: S9 Fig — Age-matched Gal4/UAS-stwl-RNAi transheterozygous virgin females were continuously mated to 1–5 day old y w males to determine the effect of stwl KD in follicle cells and germ cells. Progeny per day is the total number of progeny that emerged as in the indicated timeframe, divided by the number of days in that span. Each vial contained 10 females and 10 males. C355-Gal4, T155-Gal4, and C306-Gal4 drive expression in border cells and follicle cells from stage 9 onward (C306-Gal4 additionally drives expression in stalk cells); GR1-Gal4 drives expression in follicle stem cells of the germarium into later stages of oogenesis; bam-Gal4 drives expression in germ cells starting at the cystoblast; Mat-alpha-Gal4 drives expression in the post-GSC germline; bam-Gal4 drives expression in early and late germ cells; nos-Gal4 drives expression in GSCs; Act5c-gal4 and Tub-Gal4 drive ubiquitous expression in germline and somatic cells. (TIF) [file pgen.1010110.s009.tif]

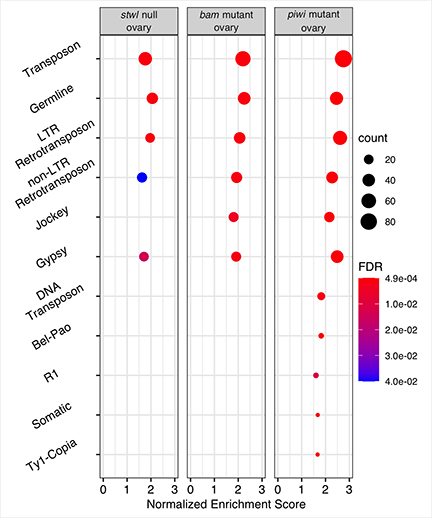

Supplement: S10 Fig — Comparison of Gene Set Enrichment Analysis (GSEA) results. Normalized Enrichment Score (NES) is plotted for each set of repetitive elements enriched among mutant/WT ovaries. Higher NES indicates that the gene set is more upregulated in mutant ovaries. Count represents the number of genes in that set. Only gene sets with FDR<0.05 are plotted. Sxl deficient ovaries were also analyzed but are not shown because they were not enriched for any repeat classes. (TIF) [file pgen.1010110.s010.tif]

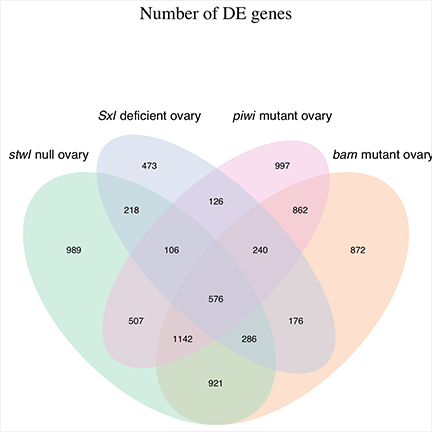

Supplement: S11 Fig — Venn diagram showing number of genes (from genomic annotation) that were upregulated (LFC>0, FDR<0.01) in each of the DESeq2 results outputs from S2 Table. (TIF) [file pgen.1010110.s011.tif]

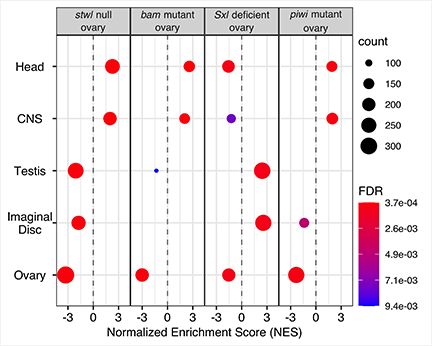

Supplement: S12 Fig — Normalized Enrichment Score (NES) is plotted for each set of tissue-enriched genes enriched among mutant or deficient/WT ovaries. Higher/lower NES indicates that the gene set is highly upregulated/downregulated in mutant or deficient ovaries. Count represents the number of genes in that set. Only gene sets with FDR<0.05 are plotted. (TIF) [file pgen.1010110.s012.tif]

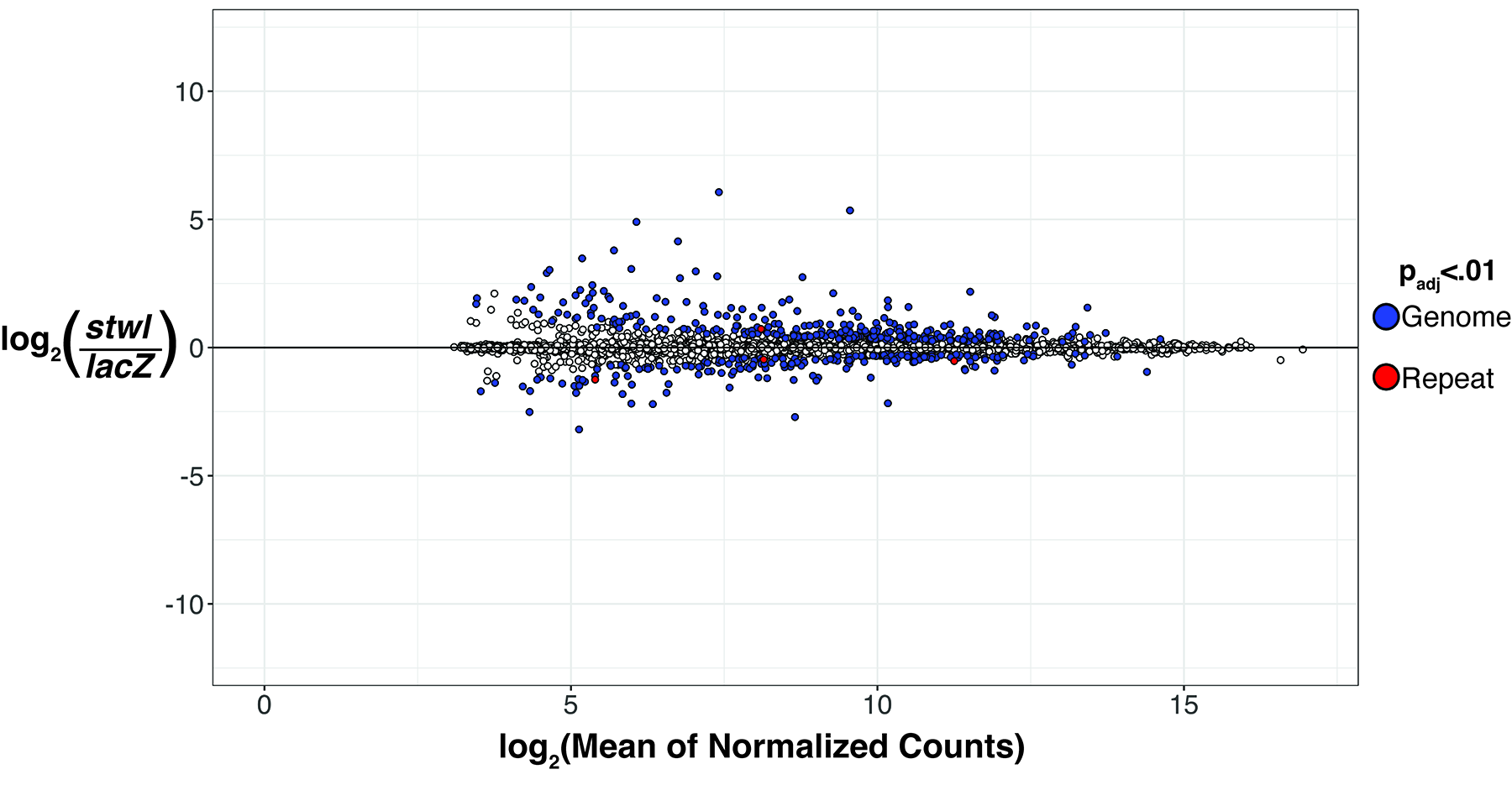

Supplement: S13 Fig — Fold-change for each gene is plotted against its mean transcript abundance across all assayed S2 cell samples (cells treated with stwl dsRNA and lacZ dsRNA as a control). Transcript abundance is represented by counts normalized according to GC-content and library size. The log2(Fold-change) values (LFC) were “shrunk” to minimize the variance associated with low-count genes. Filled points (blue and red) identify genes and repeats which are differentially expressed (adjusted p-value <0.01) in this comparison. Red points represent entries from Repbase, blue points are from the genomic annotation. Y-axis scale is identical to S8 Fig, for comparison. (TIF) [file pgen.1010110.s013.tif]

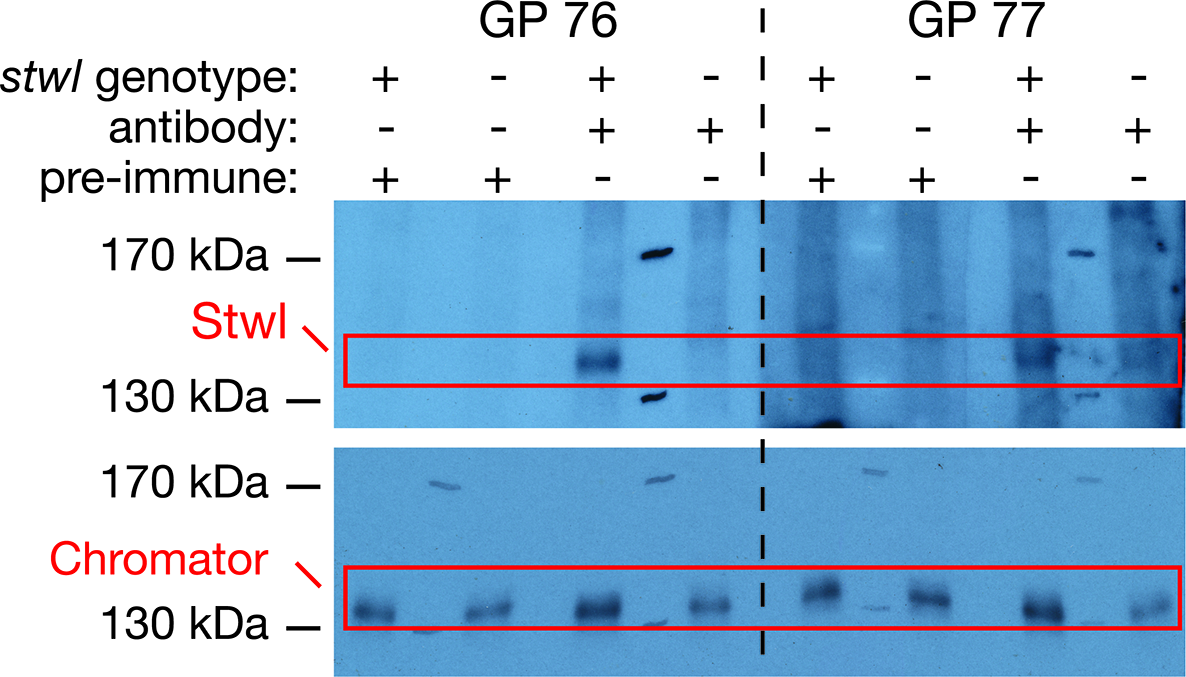

Supplement: S14 Fig — Western blots on whole-fly lysates from ~10 stwl+ (y w F10) and ~10 stwl null (stwlj6c3/Df(3L)Exel6122) individuals aged 1–4 days. 6% SDS PAGE gel was loaded with lysates as indicated (row labelled “stwl genotype”), then transferred and probed with pre-immune or antibody sera of each animal, as indicated. The bottom panel shows the same membrane stripped and re-probed with a loading control (guinea pig ɑ-Chromator). Final-bleed serum of each Stwl antibody recognizes a ~130 kDa fragment specific to stwl+ lysates. (TIF) [file pgen.1010110.s014.tif]

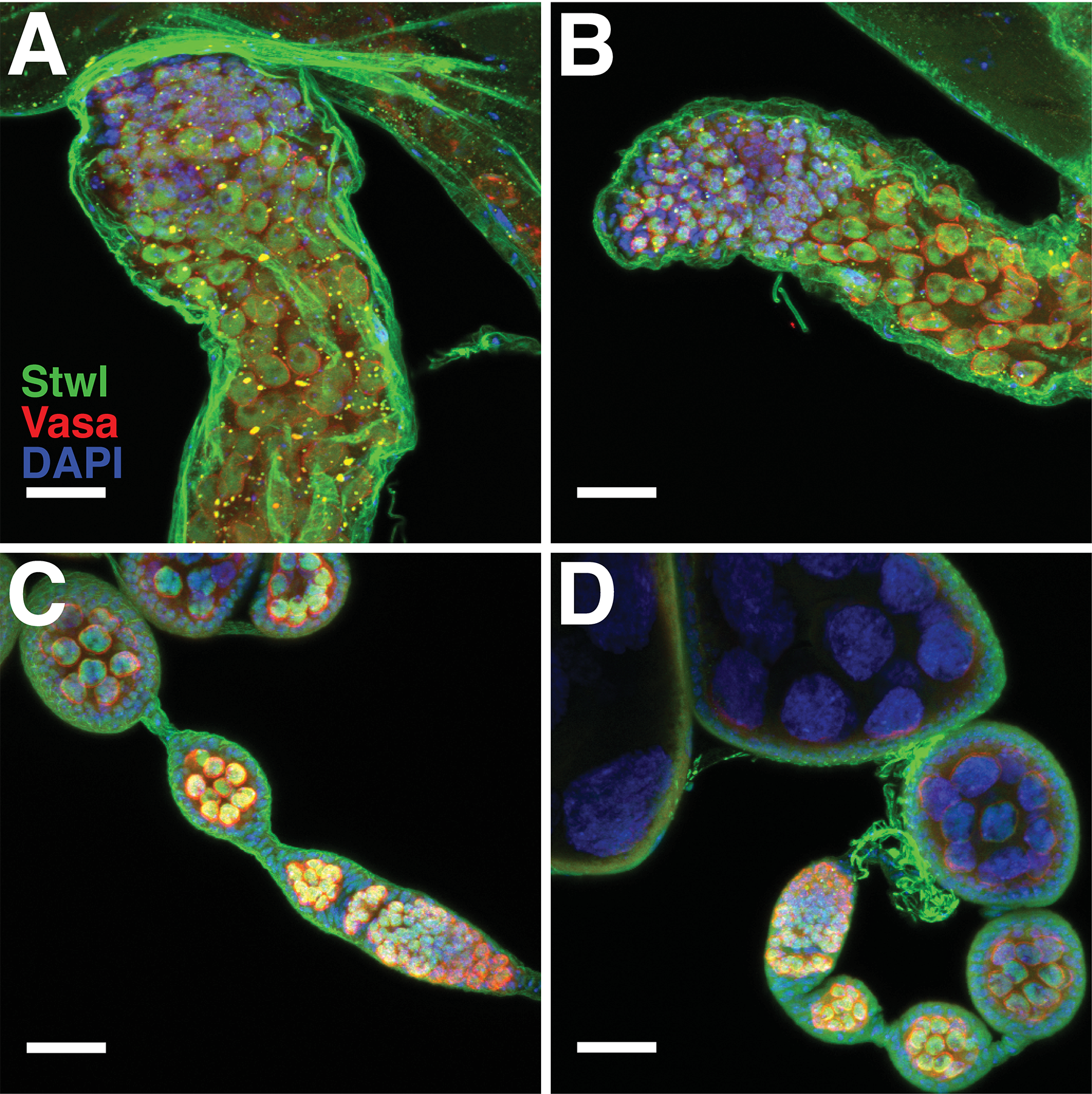

Supplement: S15 Fig — Tissues were dissected from y w F10 flies 10–15 days post-eclosion and immunostained with ɑ-Stwl sera from GP 76 (A, C) and GP 77 (B, D). Vasa labels germ cells, DAPI labels cell nuclei. All images are maximum-intensity projections from a z-series representing a depth of 10 μm. Scale bars are 20 μm. (TIF) [file pgen.1010110.s015.tif]

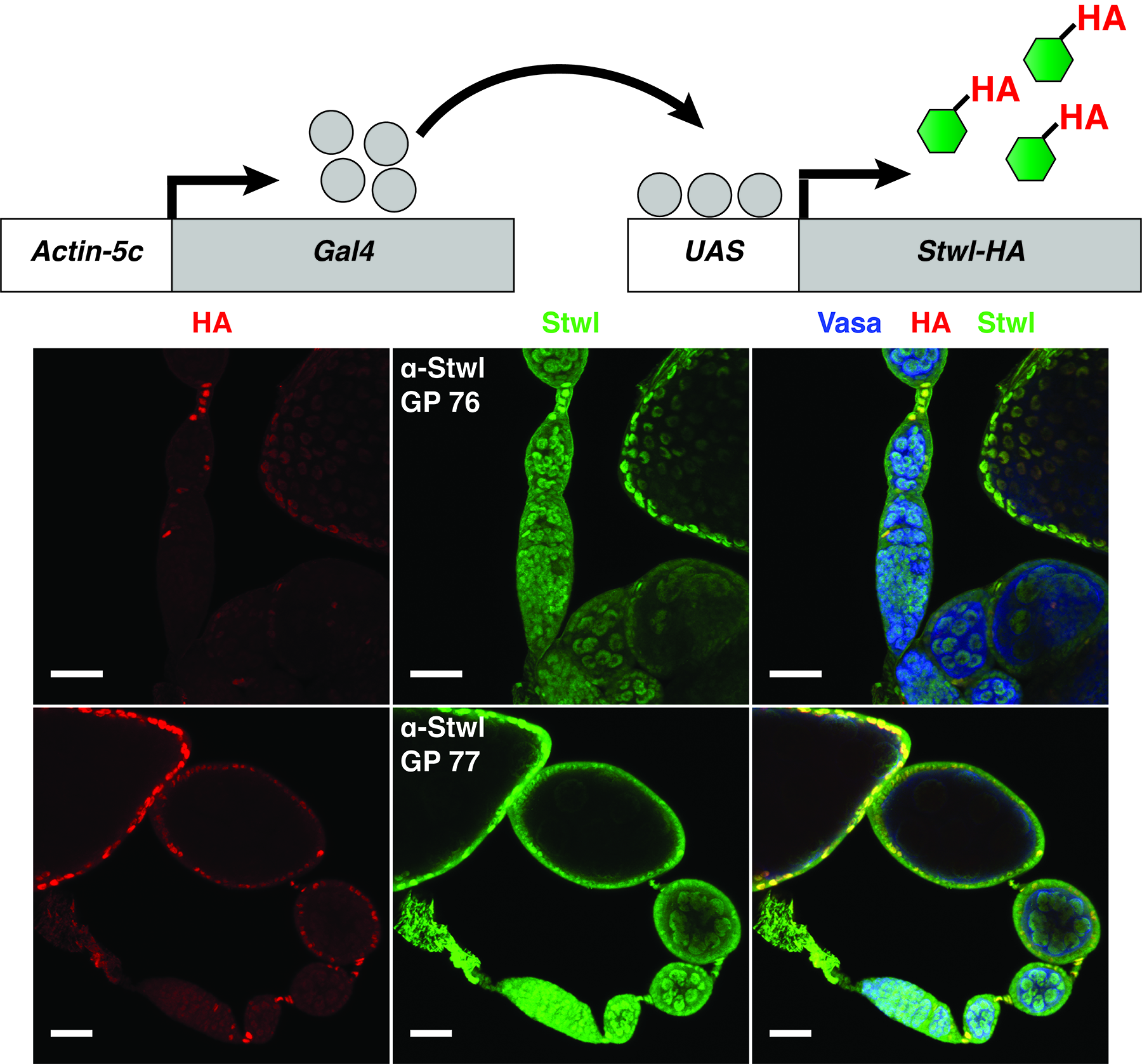

Supplement: S16 Fig — Ovaries were dissected from Act5c-Gal4/UAS-stwl-HA females 0–1 days post-eclosion. Ovaries were probed with ɑ-Vasa (germ cells), ɑ-HA, and either GP 76 or GP 77 ɑ-Stwl serum. HA signal recognizes cells in which Stwl-HA is being expressed; in these examples, expression is mostly limited to somatic cells (follicle cells and stalk cells). ɑ-Stwl signal for both antibodies clearly overlaps with HA signal, resulting in bright yellow foci in the composite image. All images are maximum-intensity projections from a z-series representing a depth of 10 μm. Scale bars are 20 μm. (TIF) [file pgen.1010110.s016.tif]

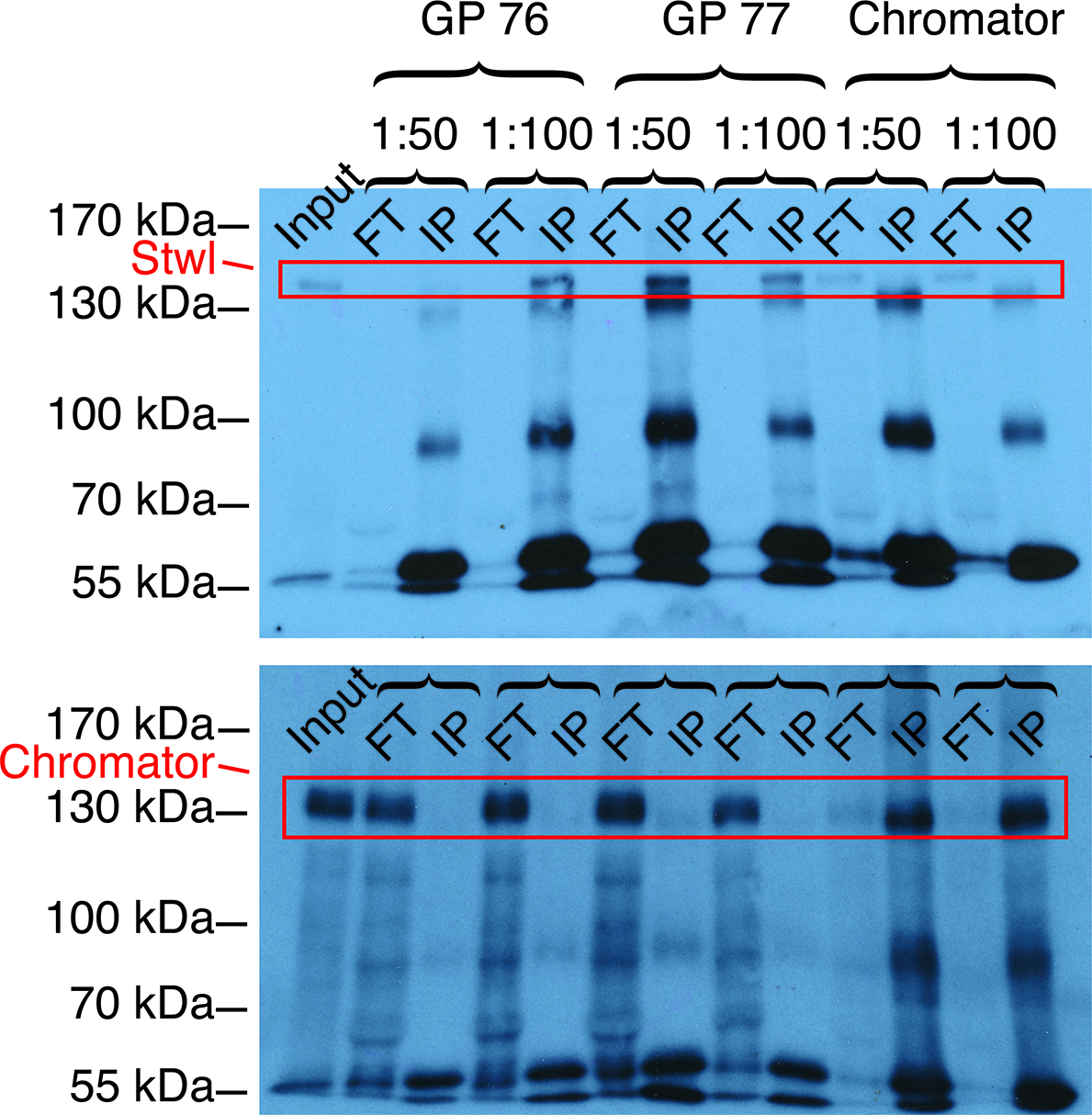

Supplement: S17 Fig — S2 cell nuclei were lysed in RIPA buffer (Input), then incubated with one of two ɑ-Stwl serum or a control antibody (ɑ-Chromator) at 1:50 and 1:100 dilutions. Antibody-Protein complexes were isolated with Protein-A Agarose beads. Western blot of input, flow-through (FT) and IP complexes (IP) probed with ɑ-Stwl GP 76 serum (top panel), then stripped and probed with ɑ-Chromator antibody (bottom panel). Stwl runs at ~130 kDa (as shown in Figs S14 and Fig 3A), as does Chromator. Both ɑ-Stwl sera immunoprecipitate Stwl effectively at a concentration of 1:100 (Stwl protein is eliminated from flowthrough). ɑ-Chromator antibody fails to immunoprecipitate Stwl (Stwl protein remains in flow-through), but successfully immunoprecipitates Chromator. (TIF) [file pgen.1010110.s017.tif]

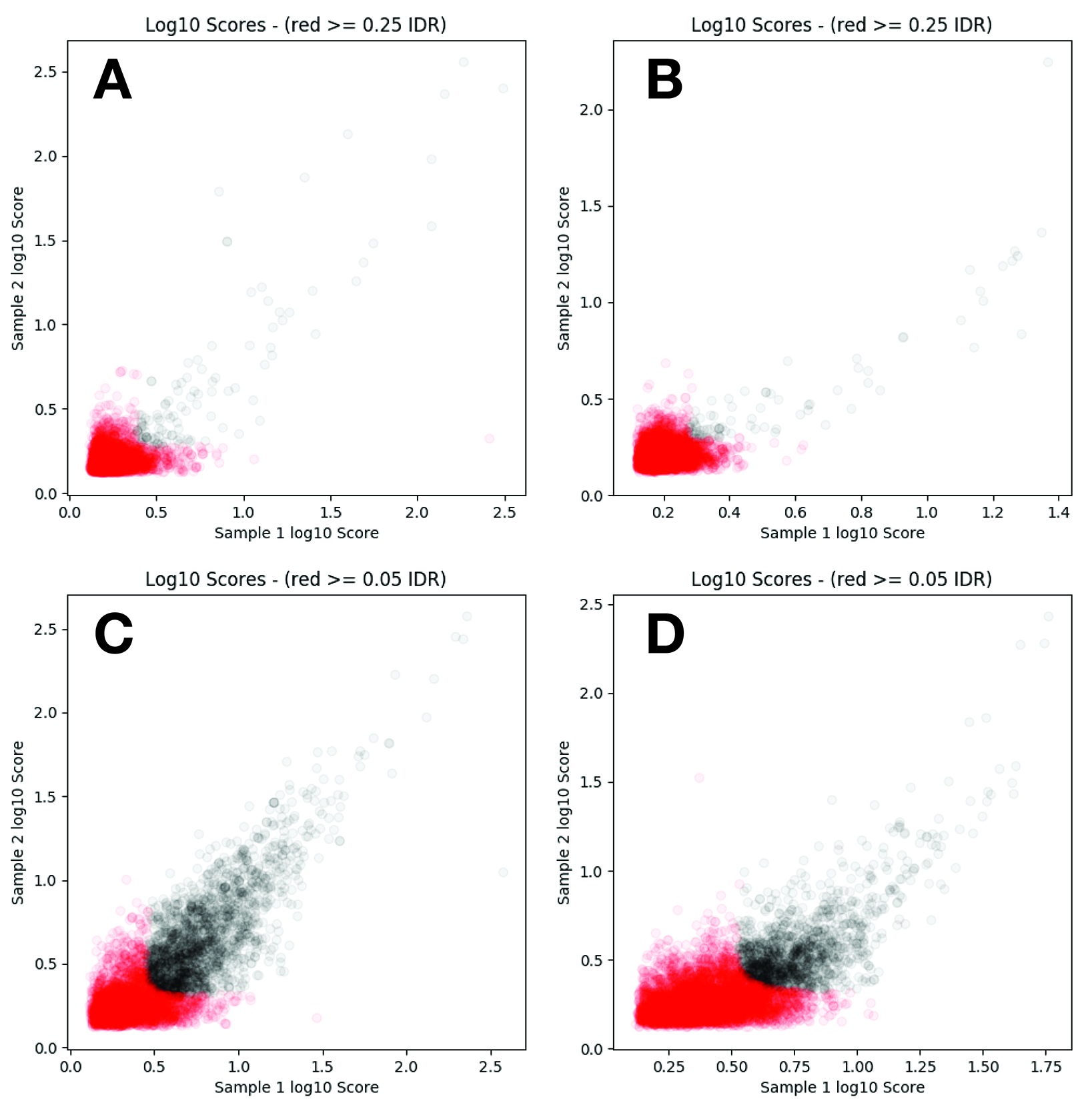

Supplement: S18 Fig — IDR plots show the distribution of peak scores in replicate 1 (x-axis) vs replicate 2 (y-axis). Grey dots are reproducible peaks that pass the given IDR threshold, red dots are irreproducible peaks. Each dot represents a ChIP-Seq peak called in both replicates of a single antibody (C, D) or mock (A, B) experiment. Peak scores reflect the fold-enrichment of reads in the IP or mock sample relative to input. IDR identifies peaks whose signal intensities (i.e. scores) are similar in both replicates. Peaks with low signal intensity in both replicates do not pass the IDR threshold, but are useful for generating a background dataset for IDR analysis. Very few peaks were identified in mock ChIP-Seq experiments, even with a relaxed IDR threshold of 0.25. (TIF) [file pgen.1010110.s018.tif]

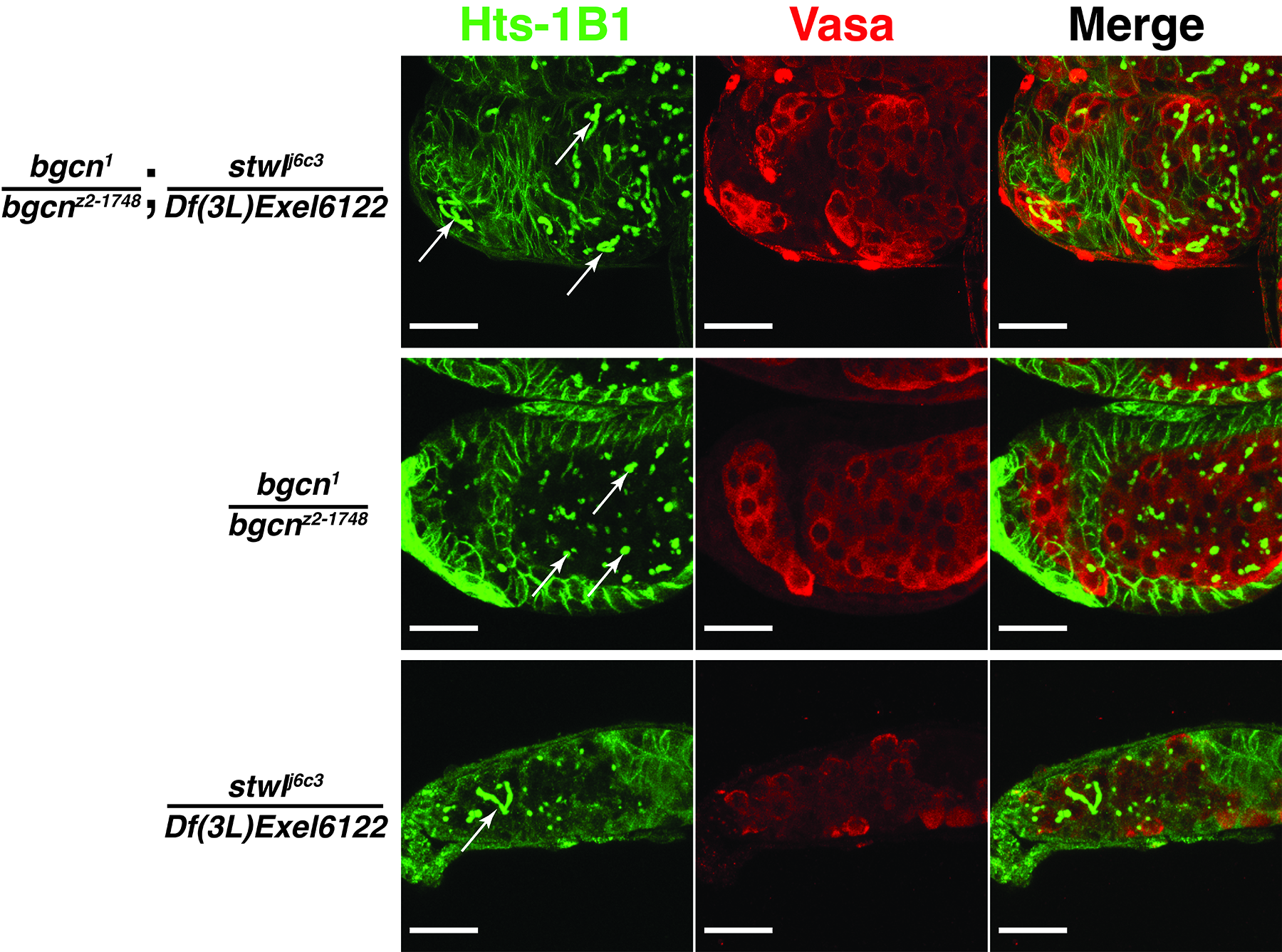

Supplement: S19 Fig — Ovaries were dissected from females of the indicated genotype 1–3 days post-eclosion. ɑ-Vasa labels germ cells, ɑ-Hts-1B1 labels branched fusomes or spectrosomes as well as follicle cell membranes. Germaria are positioned with anterior to posterior going left to right. stwl mutants form rudimentary cysts, indicated by branched fusomes, while bgcn mutant ovaries are populated with GSC-like cells, as indicated by spectrosomes and lack of branched fusomes [11–13,46–47]. Arrows point to branched fusomes in stwl mutants and bgcn; stwl double mutants, and spectrosomes in bgcn mutants. The bgcn; stwl result is consistent with previous findings [14]. All images are maximum-intensity projections from a z-series representing a depth of 10 microns. Scale bars are 20 microns. (TIF) [file pgen.1010110.s019.tif]

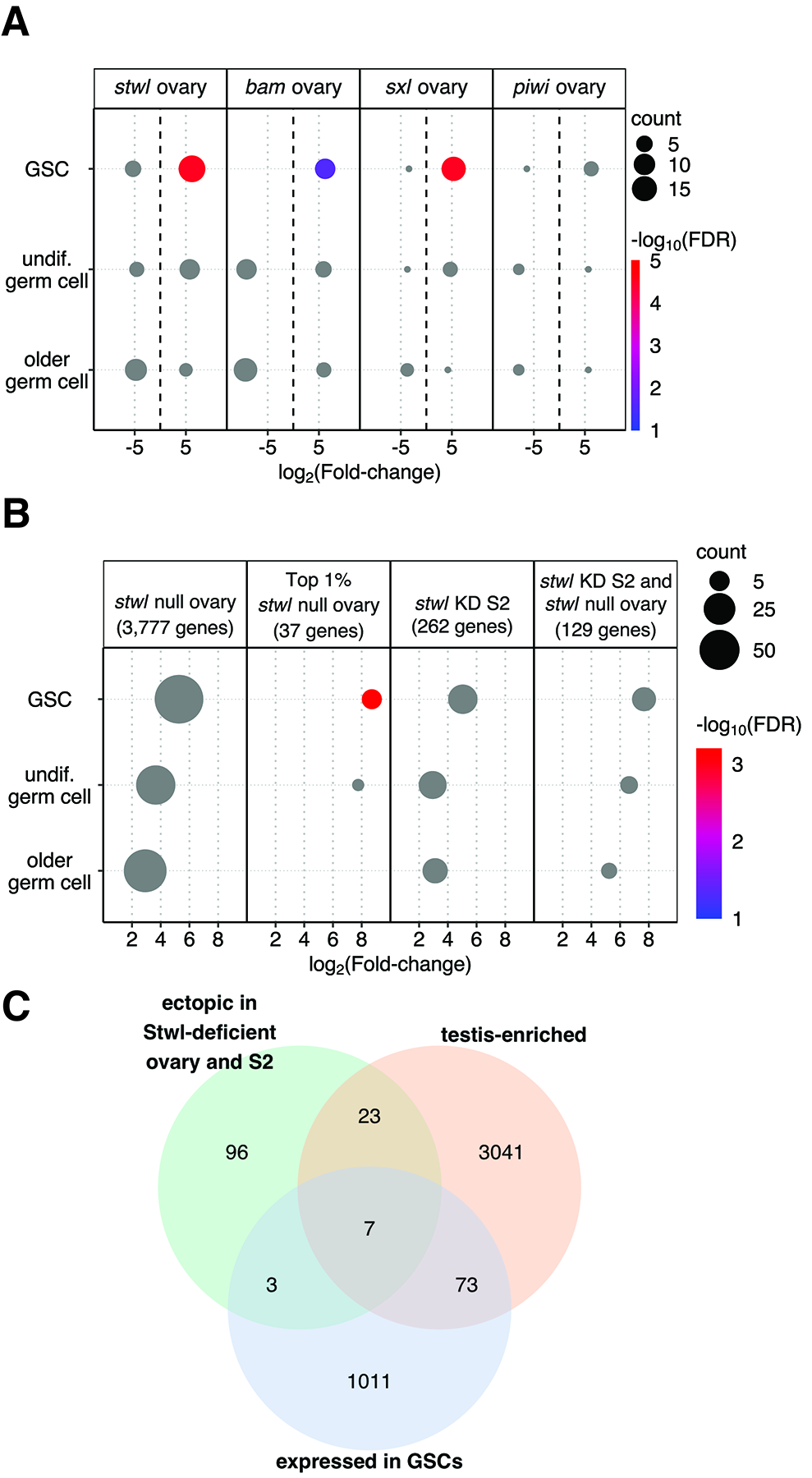

Supplement: S20 Fig — Single-cell data from Rust et al. 2020 [45] was used to identify GSC, undifferentiated germ cell and older germ cell transcripts in assayed datasets. These were the only three types of germline cells identified in the Rust et al. study. (A) Germline transcripts among the top and bottom 1% of misregulated genes in the indicated mutant or deficient ovary. (B) Germline transcripts overrepresented among ectopically expressed genes in stwl null ovaries and stwl dsRNA-treated S2 cells. Overrepresentation tests were also performed on the top 1% by LFC of ectopic genes in stwl null ovary, and of genes ectopic to both stwl null ovary and stwl dsRNA-treated S2 cells (for this intersect group, average LFC values in stwl null ovary are plotted). (A-B) Average LFC in the indicated mutant or deficient ovary is plotted for each single-cell germline cluster. All gene sets with FDR >0.1 are shaded grey. (C) Overlap of genes belonging to the following categories: enriched in testis (S7 Table), expressed in GSCs, or ectopically expressed in stwl null ovaries and stwl dsRNA-treated S2 cells. (TIF) [file pgen.1010110.s020.tif]

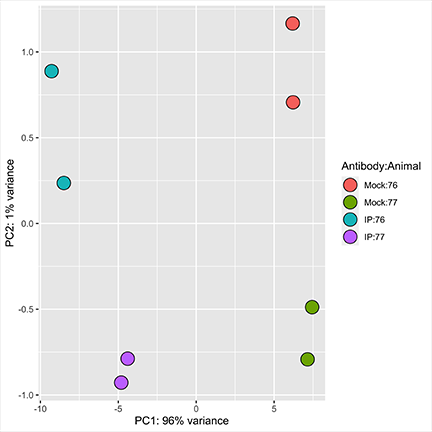

Supplement: S21 Fig — PCA for read counts generated from alignment to genomic bins and repeat index. Experiments labeled as mock were performed with pre-immune sera, IPs were performed with Stwl antibodies. Antibodies were generated from two different animals (referred to as 76 and 77) using the same epitope. DNA was isolated from two pools of S2 cells (biological replicates). The majority of the variance in the data is contained in PC1 and is explained by differences between mock and IP conditions, not by differences in the source animal or replicate pools. (TIF) [file pgen.1010110.s021.tif]
